# A real-time biphasic Kalman filter-based model for estimating human core temperature from heart rate measurements for application in the occupational field

**DOI:** 10.3389/fpubh.2024.1219595

**Published:** 2024-03-11

**Authors:** Tiziana Falcone, Simona Del Ferraro, Vincenzo Molinaro, Loredana Zollo, Paolo Lenzuni

**Affiliations:** ^1^Laboratory of Ergonomics and Physiology, Department of Occupational and Environmental Medicine, Epidemiology and Hygiene, National Institute for Insurance against Accidents at Work (INAIL), Monte Porzio Catone, Italy; ^2^Unit of Advanced Robotics and Human-Centred Technologies, Campus Bio-Medico University of Rome, Rome, Italy; ^3^Tuscany Regional Research Center, National Institute for Insurance against Accidents at Work (INAIL), Florence, Italy

**Keywords:** human core temperature, thermal strain, estimation models, wearable devices, occupational health

## Abstract

**Introduction:**

Early identification of hypothermia or hyperthermia is of vital importance, and real-time monitoring of core temperature (*CT*) of the workers exposed to thermal environments is an extremely valuable tool. From the existing literature studies, the model developed by Buller et al. in their study of 2013 that generates real-time estimates of *CT* from heart rate (*HR*) measurements using the Kalman filter (KF) shows good potential for occupational application. However, some aspects could be improved to reliably handle the existing very wide range of workers and work activities. This study presents a real-time *CT* estimation model, called the Biphasic Kalman filter-based (BKFB) model, based on *HR* measurement, with characteristics suited to application in the occupational field.

**Methods:**

Thirteen healthy subjects (six female and seven male) were included in the study to perform three consecutive tasks simulating work activities. During each test, an ingestible *CT* sensor was used to measure *CT* and a *HR* sensor to measure *HR*. The KF methodology was used to develop the BKFB model.

**Results:**

An algorithm with a biphasic structure was developed using two different models for the increasing and decreasing phases of *CT*, with the ability to switch between the two based on an *HR* threshold. *CT* estimates were compared with *CT* measurements, and with respect to overall root mean square error (RMSE), the BKFB model achieved a sizeable reduction (0.28 ± 0.12°C) compared to the Buller et al. model (0.34 ± 0.16°C).

**Discussion:**

The BKFB model introduced some modifications over the Buller et al. model for a more effective application in the occupational field. It was developed using data collected from a sample of workers (heavily weighted toward middle-aged, not very fit, and with a considerable fraction of female workers), and it also included two different modeling of *CT* (for the up- and down-phases), which allowed for better behavioral modeling in the two different stages. The BKFB model provides *CT* estimates reasonably in comparison to the measured intra-abdominal temperature values in both the activity and recovery phases but is more practical and easier to use for a real-time monitoring system of the workers' thermal states.

## 1 Introduction

There are several occupational sectors (e.g., construction and agriculture) where specific combinations of environmental thermal conditions, clothing, and metabolic activity may induce a potentially critical thermal strain for the worker, for example, those occurring in recent years due to the effects of climate change. Some of the strategies that generally can be deployed involve work organizations (1, 2), technical interventions, and the use of innovative technologies (usually wearable devices); the latter strategy attempts to mitigate the effect of exposure to thermal environments (3–6) and/or to provide real-time thermophysiological monitoring of the worker. This monitoring can be accomplished through a sensorized wearable device designed *ad hoc*, which may allow, for example, the monitoring of the worker's thermal state in order to prevent the onset of any hypothermic or hyperthermic conditions and to alert the worker when necessary. The physiological parameter that is generally used as the indicator of human thermal state is the core body temperature (*CT*), i.e., the temperature of the deep tissues of the human body core (7). A wide range of methods, including the measurement of rectal, esophageal, intra-abdominal, sublingual, tympanic membrane, external auditory canal, and urine temperature [all of which are discussed in ISO 9886:2004 (8)], are available to record a “direct” estimate of *CT*. These methods are more typically used in the clinical field or in laboratory studies and appear impractical for continuous monitoring of *CT* in workplaces due to different reasons related to the invasive nature of the measurement, worker safety, possible interference with the work activity and any personal protective equipment (PPE), and non-acceptance by workers. A potentially more practical alternative is to get an “indirect” estimate of *CT* using one or more correlated physiological parameters that can be measured without introducing appreciable discomfort for the worker. There are two main approaches available to estimate *CT* indirectly: the first is based on thermoregulation models, and the second involves the use of data-driven models.

Thermophysiological models are typically more complex because they include several equations that simulate the behavior of the various functions of the human thermoregulation system. Several models have been developed, with different degrees of complexity depending on how many body segments and nodes are considered in the model (9). These include one-node models (10, 11) that simulate the human body as a single unit, two-node models (12–15) that consider the human body to consist of two shells (i.e., core and skin), multi-node models (16–18) that assume a multilayer structure of the human body, and multi-element models (19–22) that simulate different parts of the human body with their different geometric properties, but without considering the uniform temperatures of the nodes. These models are typically employed in laboratory studies, sometimes in combination with thermal manikins (23–25). Thermophysiological models typically necessitate various input variables that must be known in advance, difficult to acquire by a wearable device, and potentially less suitable to be used in real-time mode, due to the complexity of their management.

The alternative strategy for estimating *CT* is to employ data-driven models (26). These models rely on the exploration of relationships between the inputs and outputs of a system without direct knowledge of its physiological behavior. Using regression algorithms, these models enable the estimation of the value of a variable that is not directly observable from the measurement of one or more directly observable variables. Data-driven models are more suitable than thermoregulation models for real-time monitoring of *CT* in workplaces because they require less complex mathematical operations and fewer input variables (and, consequently, fewer sensors are worn by the worker, which, in turn, generates less discomfort).

A previous systematic review (27) identified several data-driven models for estimating *CT* in real-time but only few of these models appeared suitable for integration in a wearable device such as those based on heat flux (28, 29), skin temperatures (30), heart rate (*HR*) (31–34), or a combination of these (35–38). Among these parameters, *HR* seems to be a good compromise between being the most suitable for monitoring and application in occupational contexts (its measurement does not interfere with work activities or the presence of PPE), correlating with *CT*, easy to measure (using a non-invasive sensor) and accepted by workers. Buller et al. (31) developed a model that requires only continuous monitoring of *HR* and employs a simple but very powerful mathematical method, i.e., the Kalman filter (KF) (39, 40), to calculate the step-by-step time evolution of *CT*. The model has been updated over time. Different functional forms have been explored, starting with a linear function for the observational model (41), evolving first to a quadratic function (31) and finally to a sigmoidal functional form (42, 43). The original model has been tested by Buller et al. (31, 32) in a variety of settings with regard to the environment, activity performed, acclimatization, hydration, and clothing. Estimates of *CT* show good overall agreement with measurements (31, 32), although they appear to underestimate *CT* for low work rates (31, 34) and overestimate *CT* for very high work rates (31). The model also seems to provide better predictions during the activity phase with respect to the recovery phase (44), and this is likely due to the fact that it is based on a single relationship that correlates *HR* with *CT*, whereas the latter has an inherent hysteretic nature and can follow two separate behaviors during the warm-up and cool-down phases. One challenge associated with the use of this method is that it requires an initial (*t* = 0) *CT* value. Buller et al. (32) attempted to overcome this issue by comparing the Buller et al. model (31) performance outcomes obtained using the subjects' initially measured *CT* values vs. those obtained using the initial *CT* value set at 37.1°C for all participants. The results of this study revealed that the higher accuracy provided by the initial measured *CT* values was reduced within approximately 30 min.

In general, it is difficult to extend the findings from Buller et al.'s study (31) to the worker population, because most of the participants in the study were young, athletic, and male soldiers, while a large part of the workers are less young (45) and not fit, with a considerable fraction of women as well. It is well recognized that subjective factors, such as gender, as well as anthropometry, age, and fitness, may have an impact on the thermoregulation system response (46, 47); therefore, obtaining an estimation *CT* model calibrated to the real working population is crucial. Overall, while the method proposed by Buller et al. (31) provides an unquestionably significant advancement in *CT* estimation methods, there is still potential for improvement, especially in terms of the adaptation for use in the occupational field. In this context, the present study intends to contribute to the real time *CT* estimation modeling, developing an algorithm based on two different models (biphasic model). The algorithm is specifically designed in an attempt to provide a more accurate modeling of the *CT* up- and down-phases, with the ability to switch between the two phases automatically, and obtained by data collected from a population of workers (both male and female participants) during simulations of a sequence of work activities. The biphasic model developed was based on the application of the KF to HR data, which make the model implementable in a sensorized wearable device that is suitable to be used in workplaces.

## 2 Materials and methods

### 2.1 Participants

Thirteen healthy participants (six female and seven male) were included in the study, whose mean (m) ± 1 standard deviation (SD) of the descriptive characteristics are summarized in Table 1. The participants were recruited among the employees of the National Institute for Insurance against Accidents at Work (INAIL), Research Center of Monte Porzio Catone, Italy. Participants who do not have a history of metabolic, cardiovascular, pulmonary, musculoskeletal, and gastrointestinal diseases were selected for the study. All participants gave their written informed consent, and the study was conducted according to the Declaration of Helsinki and approved by the local ethics committee (protocol number 0078009/2021).

**Table 1:** Average descriptive characteristics of the participants.

	**All participants**
n	13 (7M, 6F)
Age (year)	47 ± 4
Height (m)	1.73 ± 0.10
Weight (kg)	75.5 ± 17.7
BMI (kg/m^2^)	24.98 ± 3.35
BSA (m^2^)	1.88 ± 0.26

Data are presented as m ± 1 SD; BMI, body mass index; BSA, Body Surface Area; F, female participants; M, male participants; n, number of participants.

### 2.2 Experimental protocol

Participants were asked to abstain from smoking, drinking alcohol or coffee, and performing heavy exercise in the 24 h prior to the test. For each participant, a training section was scheduled during the days before the test, and instructions about swallowing the ingestible core body temperature sensor 3 h before arriving at the laboratory were given. Participants, each on a different scheduled test day, arrived at the laboratory at the same time in the morning in order to rule out the effects of circadian rhythms. They wore a t-shirt, shorts, and athletic shoes and were asked to abstain from drinking throughout the duration of the experiment. The height and weight of participants were collected using a professional height rod (Height Rod 5003, Soehnle Industrial Solutions, Germany) and a digital scale (Seca supra 719, Seca, USA), respectively. An ingestible core body temperature sensor (CorTemp Monitoring System, HQ Inc., Palmetto, FL, accuracy ± 0.1°C) was used to measure *CT* values every 15 s that were wirelessly transmitted to a data recorder worn by the participant at the waist level. Participants were instrumented by a heart rate sensor (Polar Electro, Finland, accuracy ± 1%) positioned at the chest using a band to measure *HR* data and connected with a wearable metabolic system (K4b2, Cosmed, Italy), which recorded cardiorespiratory data breath by breath. Before the tests, participants remained seated in a room in a thermoneutral environment (t_a_ = 23°C), allowing *CT* and *HR* to settle at the respective resting values. Once resting values were reached, participants were asked to enter the climate chamber (INAIL, Monte Porzio Catone, Italy) with an air temperature (t_a_) of 32°C, a relative humidity (RH) of 40%, and an air velocity (v_a_) of 0.3 m/s and perform the following three consecutive tasks (with a total duration of 24 min, without pause between tasks):

- Task 1 (T1): going up and down from a three-step work ladder for 6 min with a repetition frequency of 28 actions per minute (14 ascents and 14 descents);
- Task 2 (T2): lifting a 5-kg plastic crate from the ground to a standing position and returning it to the ground, for 6 min with a repetition frequency of 12 lifts per minute;
- Task 3 (T3): walking on a treadmill for 12 min at a speed of 5 km/h.

For each task, a metronome imposed the rhythm at the repetition frequency selected for the task.

After completing the tasks, participants were asked to leave the climatic chamber and sit in the room at t_a_ = 23°C to allow the parameters to reach the resting values.

### 2.3 Data analysis and signal processing

Data were processed using MATLAB software (version R2021a, MathWorks, Natick, MA, US).

*HR* and *CT* signals were synchronized. Any few missing *CT* data were reconstructed with estimates extrapolated from autoregressive fits of the remaining samples. The noise from the two signals was minimized by applying a smoothing, which uses an outlier non-influenced linear polynomial regression weight function between five neighboring data points. This method attributes a weight of zero to data outside six mean absolute deviations, excluding outliers from the smooth calculation. Both signals were averaged over consecutive time windows of 30 s.

### 2.4 Biphasic Kalman filter-based model

A model, called Biphasic Kalman filter-based (BKFB), was developed to estimate *CT* from *HR* independently during the up- and down-phases. The KF method was applied to develop the two analytical relations to model the up- and down-phases (39, 40):

- For the up-phase, the analytical relation was derived from the dataset that contained the data from all the 24 min of the activity phase that included T1, T2, and T3;
- For the down-phase, the analytical relation was obtained from the dataset that comprised data from the 19 min of the recovery period immediately following the activity phase.

As required by the KF methodology (39, 40), two sets of equations were implemented for each of the previous two phases corresponding to the time update and the measurement update. Two linear equations were derived for the time update by applying a linear regression between the *CT* values measured at time t and those measured at time t-1. The process noise (*u*) of the estimates was assumed to be a normal probability distribution [*u*~*N*(0, γ^2^)], and γ was calculated as the SD of the normal probability distribution of the Δ*CT*(*CT*_*t*_−*CT*_*t*−1_) on the time windows of 30 s. For the measurement update, 2 second-degree polynomial fits were derived employing the regression method and applied to the *HR* and *CT* data, the latter binned every 0.1°C, meaning that discretization with 0.1°C steps has been applied to the *CT* data in the *CT–HR* plane. This step implies that different *HR* values may correspond to the same detected *CT* value, and in order to obtain a univocal correspondence between *CT* and *HR* values, the *HR* values were averaged. The squared sum of error (SSE) weighted by the square of the uncertainty of each bin was minimized in the regression to yield the optimal estimation coefficients. The measurement noise (*v*) was assumed as a normal probability distribution [*v*~*N*(0, σ^2^)], and σ was computed as the mean of the SD of the *HR* values of each *CT* bin.

The BKFB model selects one of the two phases (either up or down) switching between them according to the following criteria: when at least three consecutive *HR* measurements exceed a given threshold, the model runs in the up-phase; when at least three consecutive *HR* values fall below the threshold, it runs in the down phase. The choice of the value to be assumed as the threshold was addressed by exploring different values, ranging from a minimum of 100 bpm to a maximum of 120 bpm with a step of 5 bpm, and looking for the one that minimized the total SSE, calculated as the sum of the SSE of all subjects over time. The analysis revealed that the optimal transition threshold was 115 bpm, and this value was assumed as the switching threshold. At the beginning of the estimation, this switching criterion requires three consecutive *HR* measurements and thus results in an initial three-sample lag. The initial *CT* value required by the model to derive the subsequent *CT* estimates was fixed at 37.43°C and was computed by averaging the *CT* data collected from all participants during 3 min of rest before the activity phase. The initial variance value (*v*_0_) required for estimating the variance over time was set to 0.

### 2.5 Performance evaluation

The root mean square error (RMSE) was computed to quantify the agreement between the measured and estimated values of *CT*.

### 2.6 Statistical analysis

The statistical analysis was performed using MATLAB software (version R2021a, MathWorks, Natick, MA, US).

The normality of the data was tested using the Kolmogorov-Smirnov test.

The Mann-Whitney U test was applied to detect statistical differences in the RMSE values between the BKFB model and the Buller et al. model (32) (inter-model analysis) and to compare the performance of the two models both by subject and overall (considering all subjects), with the statistical significance level set at a *p*-value of < 0.05.

## 3 Results

The results obtained in this study were presented first showing the time trends of the average measured data for *CT* and *HR*, then outlining the equations developed for the BKFB model, and finally exhibiting the comparison between the time trend of measured and estimated *CT* data [the latter by applying the Buller et al. (32) and the BKFB models] with corresponding performance outcomes.

### 3.1 Measured *HR* and *CT*

**Figure 1:**
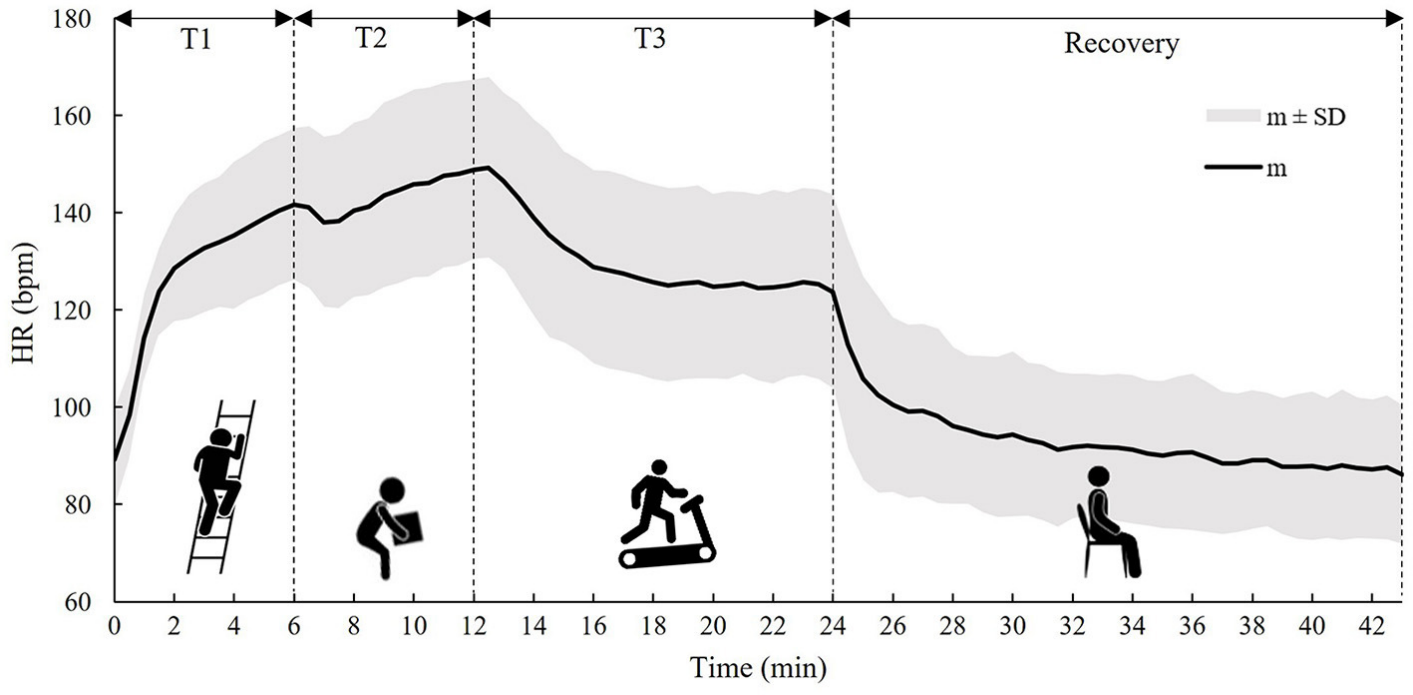
The time trend of the m ± 1 SD of the *HR* calculated on all subjects during the three tasks (T1, T2, and T3) and the recovery phase.

Figure 1 shows the time trend of the m and SD of *the HR* values measured during the activity phase (T1, T2, and T3) and the recovery phase for all 13 tested subjects.

In Figure 1, it can be observed that *HR* starts to increase immediately with the onset of T1 and continues to increase in T2, while, with the onset of T3, *HR* starts to decrease and then stabilizes around the 6th min due to the lower metabolic activity compared to T2. In the recovery phase, an exponential trend toward pre-activity conditions was observed.

**Figure 2:**
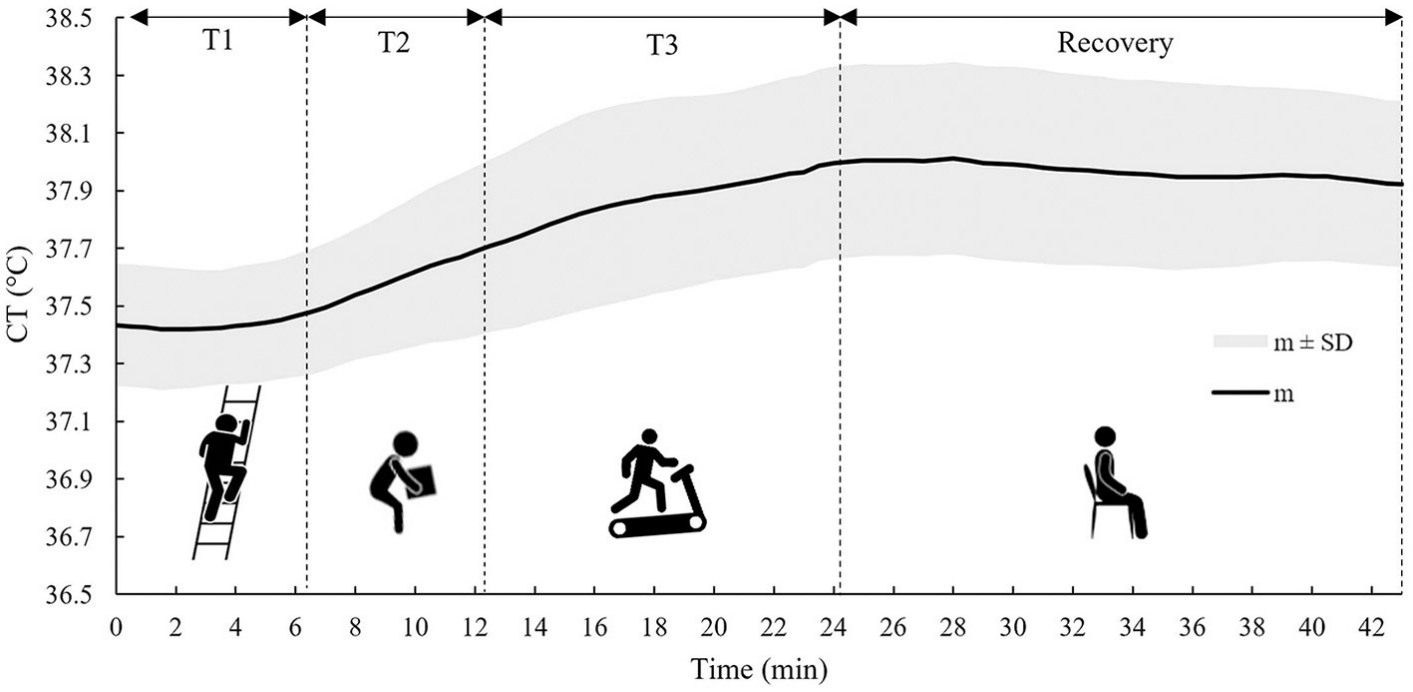
The time trend of the m ± 1 SD of the *CT* calculated on all subjects during the three tasks (T1, T2, and T3) and the recovery phase.

Figure 2 shows the time trend of m and SD of *CT* values measured by the ingestible core body temperature sensor during the activity phases (T1, T2, and T3) and the recovery phase for all 13 tested subjects.

As can be noticed from Figure 2, *CT* showed a much slower response compared to *HR*; in fact, *CT* time course was approximately steady during T1, and it only started to increase in T2. In T3, it increased over time and then stabilized during the recovery phase, showing a very gradual decline.

### 3.2 Biphasic Kalman filter-based model

In both the up- and down-phases of the model, the same equation was obtained for the *CT* time update and is as follows (Eq. 1):


(1) $$\begin{array}{l} {CT^{\prime }}_{t}=\;CT_{t-1} \end{array}$$

The regression coefficients were 0.9997 (determination coefficient, R^2^ = 1.00) and 1.0001 (R^2^ = 1.00) for the up- and down-phases of the model, respectively, and were then approximated to 1 for both phases.

**Figure 3:**
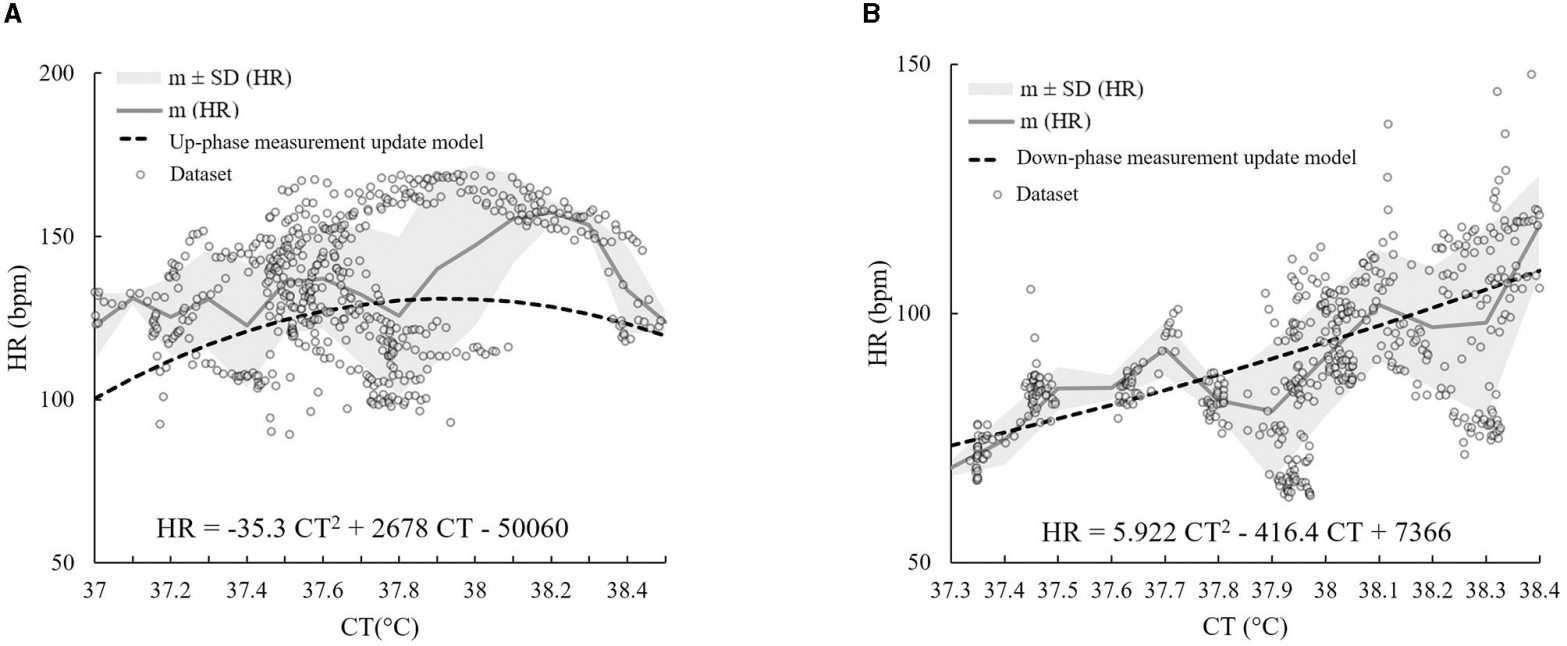
The BKFB model: **(A)** The up-phase measurement update model with the corresponding datasets and m ± 1 SD of **HR** values corresponding to each **CT** bin of 0.1°C; **(B)** The down-phase measurement update model with the corresponding datasets and m ± 1 SD of **HR** values corresponding to each **CT** bin of 0.1°C.

For the up-phase of the model, γ was 0.016, while for the down-phase of the model, γ was 0.011. The two measurement update models of both the up- and down-phases, with the corresponding datasets from which the model was developed, are shown in Figure 3. The two graphs also include m and SD of *HR* values corresponding to each *CT* bin of 0.1°C.

The regression equation obtained for the up-phase measurement update model is given as follows (Eq. 2):


(2) $$\begin{array}{l} HR=-35.3\;CT^{2}+2678\;CT-50060 \end{array}$$

The respective R^2^ value was 0.46, while the regression equation for the down-phase measurement update model is as follows (Eq. 3):


(3) $$\begin{array}{l} HR=5.922\;CT^{2}-416.4\;CT+7366 \end{array}$$

The R^2^ value was 0.72.

For the up-phase, σ was 14.081, while for the down-phase, it was 8.692.

### 3.3 Measured vs. estimated core temperatures

*CT* estimates obtained by using the BKFB model were compared with the *CT* data measured with the ingestible core body temperature sensor and the *CT* estimates obtained by the Buller et al. model (32) on the same dataset, where the latter implemented a monophasic quadratic function and used 37.1°C as the initial *CT* value (t = 0). The comparisons were performed both by subject and considering all subjects (overall).

#### 3.3.1 Comparisons by subject

**Figure 4:**
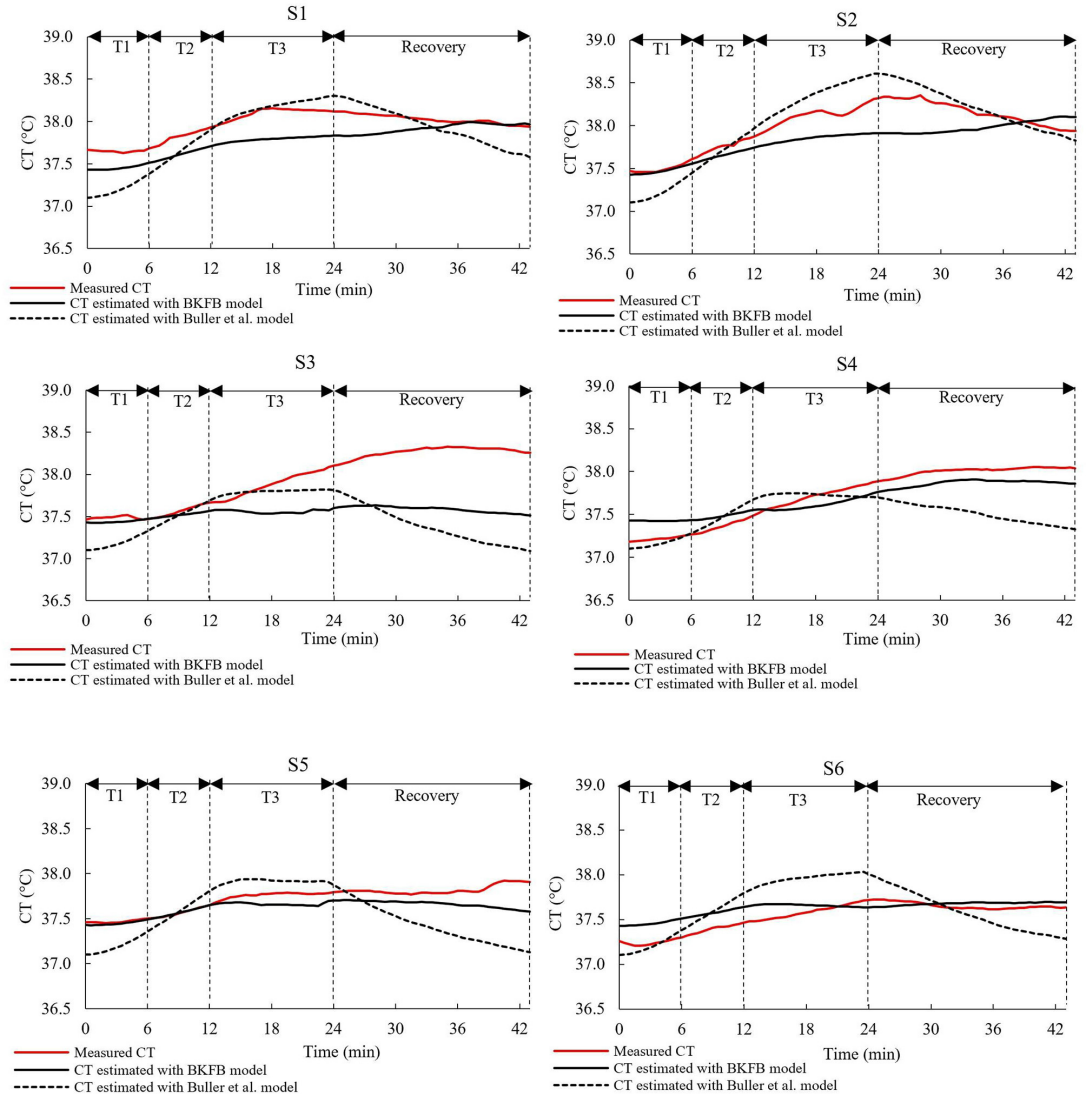
The time trends of the measured *CT*, the *CT* estimated by the BKFB model, and the *CT* estimated by the Buller et al. (32) model for subjects 1 to 6.

**Figure 5:**
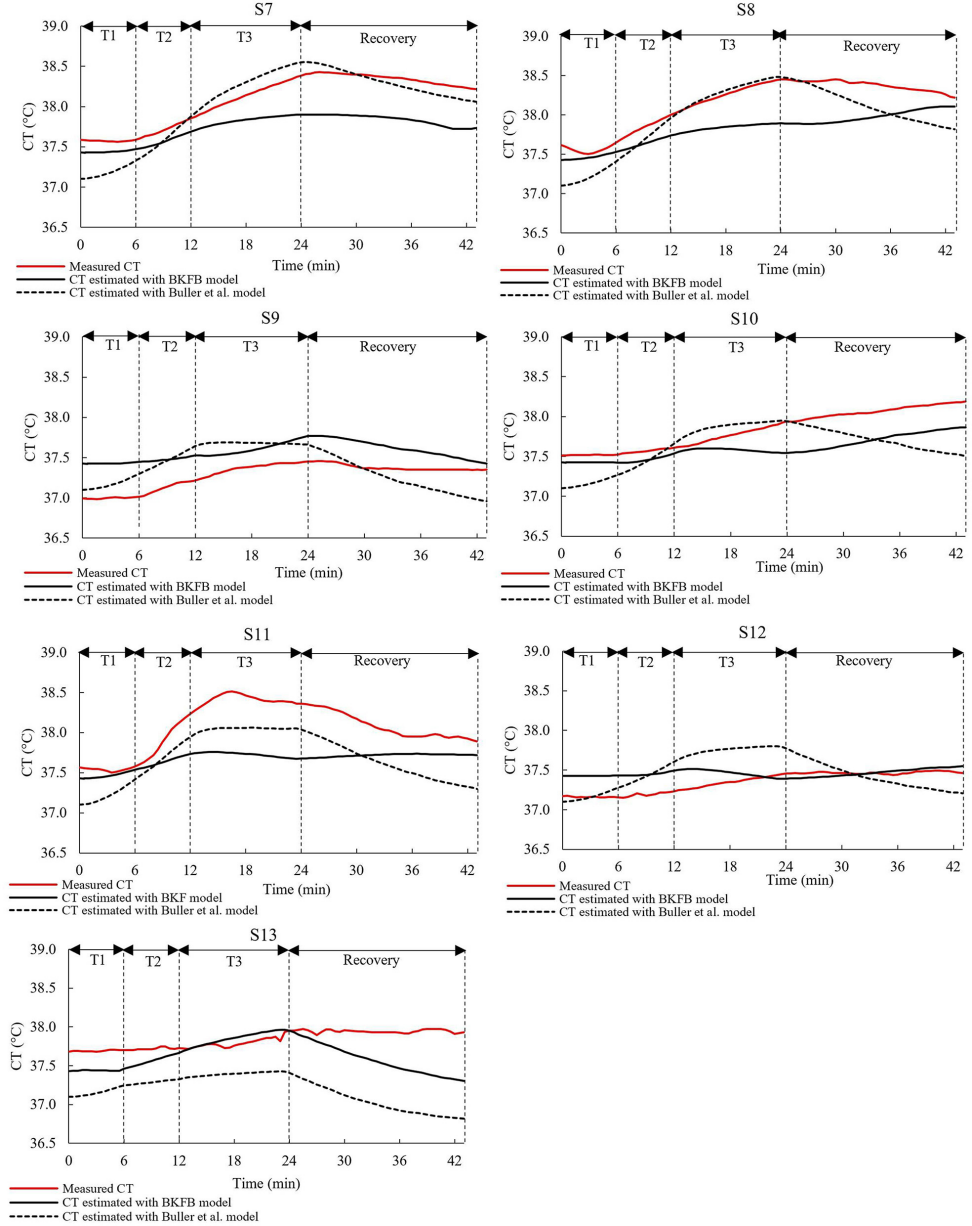
The time trends of the measured *CT*, the *CT* estimated by the BKFB model, and the *CT* estimated by the Buller et al. (32) model for subjects 7 to 13.

Figures 4, 5 show, for each tested subject, the time trend of the *CT* as follows: (a) measured by using the ingestible core body temperature sensor; (b) estimated by the BKFB model; and (c) estimated by the Buller et al. model (32).

From the graphs in the two panels (Figures 4, 5), it can be observed that the BKFB model estimates *CT* time trend reasonably well for most subjects, following their *CT* behaviors over time. In 10 cases (S1, S2, S3, S4, S5, S7, S8, S10, S11, and S13), the BKFB model seemed to slightly underestimate the time trend of the corresponding measured *CT* for the majority of the time, while in three cases, the model seemed to slightly overestimate it (S6, S9, and S12) for most of the time. To quantify the subject-by-subject agreement between measured *CT* values and those estimated by the BKFB model and the Buller et al. model (32), the RMSE values were calculated over the entire duration of the test (RMSE_total_) and for the activity phase (RMSE_activity_) and the recovery phase (RMSE_recovery_) and are listed in Table 2.

**Table 2:** RMSE and *p*-values calculated for subject-by-subject.

**Subjects**	**The BKFB model**			**The Buller et al**. **(****32****)** **model**			***p*** **-values**		
	**RMSE**_total_	**RMSE**_activity_	**RMSE**_recovery_	**RMSE**_total_	**RMSE**_activity_	**RMSE**_recovery_	**Total**	**Activity**	**Recovery**
S1	0.22 ± 0.04	**0.26** **±0.03**	**0.15** **±0.03**	0.23 ± 0.08	0.26 ± 0.10	0.19 ± 0.04	0.144	^*^	^*^
S2	0.23 ± 0.06	**0.20** **±0.04**	0.27 ± 0.07	0.19 ± 0.04	0.23 ± 0.05	**0.13** **±0.02**	0.160	^*^	^***^
S3	0.49 ± 0.23	0.24 ± 0.08	**0.69** **±0.11**	0.63 ± 0.50	0.19 ± 0.05	0.92 ± 0.43	0.436	0.68	^***^
S4	**0.15** **±0.01**	0.15 ± 0.02	**0.15** **±0.01**	0.36 ± 0.16	**0.11** **±0.01**	0.52 ± 0.15	^*^	^**^	^***^
S5	**0.13** **±0.03**	**0.07** **±0.01**	**0.18** **±0.03**	0.34 ± 0.16	0.19 ± 0.03	0.46 ± 0.20	^***^	^***^	^***^
S6	**0.13** **±0.02**	**0.16** **±0.02**	**0.06** **±0.00**	0.25 ± 0.06	0.29 ± 0.06	0.20 ± 0.04	^***^	^***^	^***^
S7	0.39 ± 0.11	0.24 ± 0.06	0.51 ± 0.01	**0.19** **±0.06**	0.21 ± 0.07	**0.11** **±0.01**	^***^	0.81	^***^
S8	0.37 ± 0.11	0.32 ± 0.09	0.43 ± 0.11	**0.25** **±0.07**	**0.20** **±0.06**	**0.29** **±0.07**	^***^	^***^	^***^
S9	0.30 ± 0.05	0.33 ± 0.06	0.25 ± 0.03	**0.26** **±0.05**	0.29 ± 0.05	**0.21** **±0.05**	^*^	0.12	^*^
S10	0.27 ± 0.06	0.16 ± 0.04	0.36 ± 0.02	0.32 ± 0.12	0.22 ± 0.05	0.41 ± 0.15	0.81	0.09	0.88
S11	0.46 ± 0.20	0.51 ± 0.23	**0.41** **±0.15**	0.40 ± 0.09	0.33 ± 0.06	0.47 ± 0.08	0.97	0.13	^**^
S12	**0.17** **±0.03**	**0.22** **±0.03**	**0.05** **±0.00**	0.26 ± 0.07	0.31 ± 0.07	0.18 ± 0.03	^***^	^***^	^***^
S13	**0.30** **±0.12**	**0.16** **±0.03**	**0.41** **±0.14**	0.70 ± 0.39	0.45 ± 0.06	0.92 ± 0.31	^***^	^***^	^***^

Values are presented as m ± SD computed for each subject by averaging the values for the duration of the phase considered. RMSE_activity_, Root mean square error calculated considering the activity phase, in °C; RMSE_recovery_, Root mean square error calculated considering the recovery phase, in °C; RMSE_total_, Root mean square error calculated considering both the activity and recovery phases, in °C; S, Subject. ^*^p < 0.05, ^**^p < 0.01, ^***^p < 0.001 according to Mann-Whitney U test.

Based on the RMSE values reported in Table 2, three classes of agreement between the measured *CT* values and those estimated by models were assumed: high agreement with RMSE_total_ ≤ 0.30°C, moderate agreement with 0.30°C <RMSE_total_ <0.40°C, and poor agreement with RMSE_total_ ≥ 0.40°C. With this assumption, an intra-model comparison revealed that the BKFB model showed 9 out of 13 subjects in high agreement, two (S7 and S8) in moderate agreement, and two (S3 and S11) in poor agreement, while the Buller et al. model (32) showed seven subjects in high agreement, three (S4, S5, and S10) in moderate agreement, and three (S3, S11, and S13) in poor agreement.

Table 2 shows the statistically significant differences in the RMSE values between the BKFB model and the Buller et al. model (32), by subject, for the three phases considered. Among the significant differences detected, the lowest RMSE value between the two models is highlighted in bold in Table 2. According to an inter-model comparison based on these values, the BKFB model obtained five out of eight (statistically significant) lowest RMSE values for the total phase [three out of eight for the Buller et al. model (32)], six out of eight for the activity phase [two out of eight for the Buller et al. model (32)], and eight out of twelve for the recovery phase [four out of twelve for the Buller et al. model (32)] showing progress associated with the BKFB model.

#### 3.3.2 Comparisons considering all subjects

**Figure 6:**
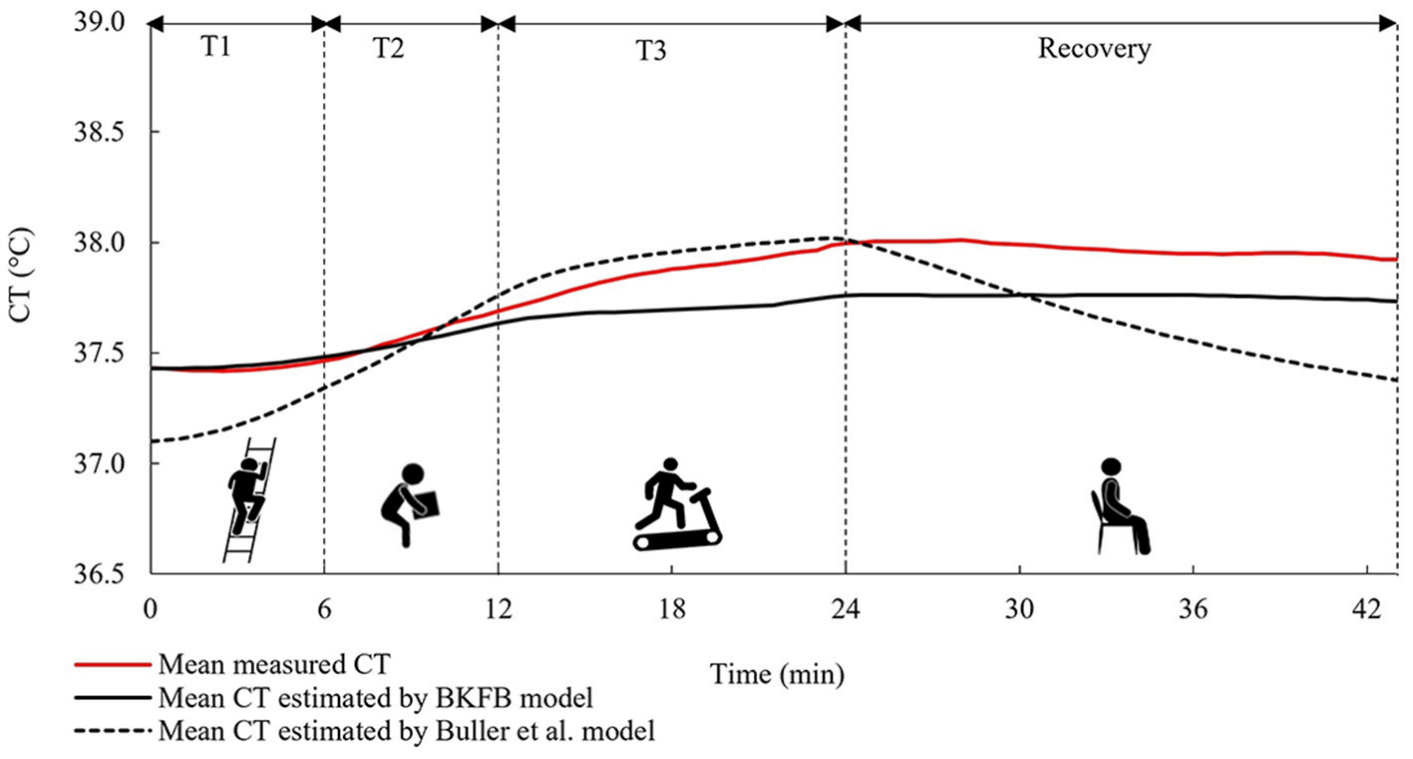
The time trends of the mean measured *CT*, the mean *CT* estimated by the BKFB model, and the mean *CT* estimated by the Buller et al. (32) model considering all subjects.

Figure 6 shows the time trend of the mean values (all subjects) of the measured and estimated *CT* [by both BKFB and Buller et al. (32) models].

The mean trend obtained by the BKFB model showed similar behavior to the mean trend of measured *CT* in all the phases monitored, remaining below it from around the middle of T2.

The overall agreement between the measured and estimated values of *CT* [by both BKFB and Buller et al. (32) models] was also quantified using the overall RMSE values, which were calculated by averaging the RMSE over all tested subjects (Table 3). Three different overall RMSE values were calculated by taking averages, namely, (a) over the entire duration of the test (Overall RMSE_total_); (b) over the activity phase only (Overall RMSE_activity_); and (c) over the recovery phase only (Overall RMSE_recovery_), and the inter-model differences were tested by the Mann-Whitney U test, as shown in Table 3.

**Table 3:** Overall RMSE and *p*-values.

	**The BKFB model**	**The Buller et al. model (32)**	***p*-values**
Overall RMSE_total_	0.28 ± 0.12	0.34 ± 0.16	0.40
Overall RMSE_activity_	0.23 ± 0.11	0.25 ± 0.08	0.47
Overall RMSE_recovery_	0.30 ± 0.19	0.39 ± 0.27	0.46

Values are presented as m ± SD computed by averaging the values for the duration of the phase and for all subjects. RMSE_activity_, Root mean square error calculated considering the activity phase, in °C; RMSE_recovery_, Root mean square error calculated considering the recovery phase, in °C; RMSE_total_, Root mean square error calculated considering both the activity and recovery phases, in °C.

As can be noted in Table 3, an inter-model comparison between the BKFB model and the Buller et al. model (32) showed that the BKFB model recorded lower RMSE values than the Buller et al. model (32) for all the phases considered, even if the statistical analysis applied does not reveal significant differences between the two models.

## 4 Discussion

The present study proposes a biphasic model for real-time *CT* estimation to be implemented in a sensorized wearable device with the aim of providing thermophysiological monitoring of a worker during his work activity. The choices made to obtain the BKFB model are the result of an attempt to find the right compromise between the need for a reasonably acceptable accuracy of the *CT* estimations, a suitable computational complexity to be managed, and the non-invasiveness of the system. With this approach, the BKFB model has many strengths, such as: (a) being obtained from a sample of workers that is around middle-age, not very fit and includes both male and female workers; (b) being based on the *HR*, a physiological parameter that can be easily and continuously measured in a non-invasive way and is generally accepted by the workers; (c) using the KF which is suitable for real-time data processing; (d) being an algorithm that uses two different models to estimate the *CT* up and down-phases, all aspects that will be discussed in the following.

The use of the *HR* value alone in the estimation of *CT* makes the monitoring system simple, due to the HR sensor that can be easily worn by workers without causing interference with work activity or discomfort to the worker. *CT* and *HR*, however, behave differently in response to the activities performed (44), and in fact, as observed from Figures 1, 2, these two parameters react differently during the three tasks and the recovery phase. In particular, *HR* responds faster to changes in activity than *CT*, because the response of the latter is delayed by the large thermal inertia of the human body (44, 48). Nevertheless, *HR* appears to be a good predictor of *CT* (31, 41). The results obtained in this study suggest that the agreement between *HR* and *CT* is quite strong in the down-phase (R^2^ = 0.72), while it is more limited in the up-phase (R^2^ = 0.46). One of the potential solutions to improve the model's predictive ability could be to use more predictor variables (such as heat flux and skin temperature measured in various parts of the body) in combination with *HR* (36, 37). However, the use of more variables in the model implies a more complex handling of a real-time monitoring system. In addition, the worker has to wear several sensors, and this can cause inconvenience to the workers. Thus, the gain in performance may not justify the possible loss of monitoring practicality.

The choice of using the KF methodology to estimate *CT* from *HR* seems to be appropriate because this offers a powerful and fast recursive computational method, which is ideal for real-time data processing. Two functional forms were preliminarily explored for the development of the two measurement update models (in the up- and down-phases of *CT*) as required by the KF: a second-degree polynomial function [proposed in the study of Buller et al. (31, 32)] and a sigmoidal functional form [proposed by Looney et al. (42) as an updated version of the Buller et al. model (31) to increase the accuracy of the lowest *CT* estimates]. The application of the monophasic sigmoidal model (42) to the dataset of this study resulted in a slightly lower overall RMSE_recovery_ (0.30 ± 0.20°C) than that obtained by applying the Buller et al. model (31) (quadratic monophasic function) to the same dataset, and a marginally higher overall RMSE_activity_ (0.29 ± 0.08°C). Because modeling the activity phase with the sigmoidal function was not performant and modeling the recovery phase was only marginally better, it was consequently decided to adopt the quadratic biphasic function for the BKFB model and to use the Buller et al. quadratic monophasic model (32) as the alternative reference method (in addition to measured *CT*) for comparisons with the model obtained in this study.

The BKFB model achieved very promising results in terms of the overall RMSE values during the activity phase (0.23 ± 0.11°C), during the recovery phase (0.30 ± 0.19°C), and when considering both (0.28 ± 0.12°C). The overall RMSE values obtained by the application of the BKFB model were lower than those obtained by the Buller et al. model (32) indicating better estimates of the *CT* data measured in this study. The RMSE_total_, RMSE_activity_, and RMSE_recovery_ were also calculated subject-by-subject in this study. The BKFB model in the recovery phase outperformed the Buller et al. model (32) in more cases compared to the activity phase, suggesting that the former likely modeled the recovery phases better. This finding is presumably because the Buller et al. model (32) used the same equation for both the activity and recovery phases, whereas the BKFB model uses an equation that is especially designed to shape the recovery phases. The lower number of cases in which the BKFB model outperformed the Buller et al. model (32) in the activity phase may depend on the limited dataset with a not very extensive *CT* range and the use of an initial estimated *CT* instead of the measured *CT*. Indeed, a big challenge in using the KF methodology for *CT* estimation is that an accurate *CT* value at t = 0 is required. In this study, it was decided to estimate the initial *CT* value by averaging the *CT* data collected from all participants during the 3 min rest period prior to the activity phase in order to obtain a more representative value of the workers' population considered. The initial *CT* value, on the other hand, may vary depending on the subjective characteristics (46, 47), and this subject-to-subject difference is also highlighted by the time courses of the measured *CT* of the 13 selected subjects (Figures 4, 5). In any case, the necessity to provide the model with the most accurate *CT* value to use at t = 0 is an ongoing common issue (32, 44), and more in-depth investigations on the quantification of the impact of the initial *CT* value on subsequent estimates are needed.

In general, the BKFB model appears to have the potential to be applied for real-time *CT* estimation in the occupational field. However, a limitation may be attributed to the not very wide range of *CT* values in the dataset that most likely made the determination of the measurement update models more challenging and may limit the predictive capabilities of the BKFB model to the *CT* values up to 38°C. A larger sample size (composed of a wider and more diversified population of workers in terms of age, anthropometry, and physical fitness) could improve the estimation and could also allow the development of age- or gender-specific models so as to make it oriented to the individual target groups. Future investigations should focus on testing the performance of the BKFB model in a wider range of stressful situations, with respect to (a) other work activities performed in real workplaces (considering also static work); (b) multi-layer clothing, possibly also including PPE; and (c) additional environmental thermal conditions, both warmer and colder conditions than those explored in this study. Effects due to psychological stress shall also be addressed. Estimation models should evolve toward progressive customization (i.e., taking into account age, gender, and other variables) as a contribution to future research.

## 5 Conclusion

This study focuses on the development of a real-time core temperature (*CT*) estimation model for application in the occupational field. Employing two separate phases, one designed specifically for the *CT* up-phase and the other for the *CT* down-phase, with the ability to switch between the two, a model was developed that leverages the Kalman filter (KF) to estimate *CT* from *HR* measurements.

The Biphasic Kalman filter-based (BKFB) model obtained introduces some modifications over the Buller et al. model (31) for a more effective application in the occupational field. In particular, the BKFB model was developed from *CT* and *HR* data acquired on a more realistic sample of workers (which is heavily weighted toward middle-aged, not very fit, and with a considerable part of female workers) while simulating real work activities. This new model also includes two different modeling of *CT*, namely, one for the increasing phase and the other for the decreasing phase, allowing for better behavioral modeling of the *CT* in the two different stages. The BKFB model only requires *HR* measurement to run, which is measured with a non-invasive sensor that can be used for extended periods, without disturbing the worker. In addition to being easily used in workplaces, the model found in this study performed well (overall RMSE_total_ = 0.28 ± 0.12°C, overall RMSE_activity_ = 0.23 ± 0.11°C, and overall RMSE_recovery_ = 0.30 ± 0.19°C) when its *CT* estimates were compared to *CT* measurements obtained from the ingestible core body temperature sensor. The BKFB model also achieved lower overall RMSE values than the Buller et al. model (32) (overall RMSE_total_ = 0.34 ± 0.16°C, overall RMSE_activity_= 0.25 ± 0.08°C, and overall RMSE_recovery_= 0.39 ± 0.27°C). Despite the *CT* application range of up to 38°C, it can be concluded that the BKFB model appears to be a suitable solution to be integrated into a real-time monitoring system of the workers' thermal state due to its good performance and its ease of application and management. Future studies should aim to extend the application range of the *CT* in order to model more stressful conditions.

## Data availability statement

The raw data supporting the conclusions of this article will be made available by the authors, without undue reservation.

## Ethics statement

The studies involving humans were approved by Local Ethics Committee (protocol number 0078009/2021). The studies were conducted in accordance with the local legislation and institutional requirements. The participants provided their written informed consent to participate in this study.

## Author contributions

SDF, TF, and PL: methodology. SDF and TF: visualization. TF and PL: data curation, formal analysis, and software. VM: funding acquisition, project administration, resources, and supervision. SDF, TF, PL, and VM: conceptualization, investigation, validation, and writing of the original draft. All authors contributed to the revision of the manuscript, read, and approved the submitted version.
